# Artificial Grassland Had Higher Water Use Efficiency in Year with Less Precipitation in the Agro-Pastoral Ecotone

**DOI:** 10.3390/plants12061239

**Published:** 2023-03-09

**Authors:** Kun Zhao, Yan Qu, Deping Wang, Zhongkuan Liu, Yuping Rong

**Affiliations:** 1 College of Grassland Science and Technology, China Agricultural University, Beijing 100193, China; 2 Institute of Agro-Resource and Environment, Hebei Academy of Agriculture and Forestry Sciences, Shijiazhuang 050051, China

**Keywords:** land use type, evapotranspiration, dry matter accumulation, soil moisture, soil nutrient

## Abstract

Improving plant water use efficiency is a key strategy for the utilization of regional limited water resources as well as the sustainable development of agriculture industry. To investigate the effects of different land use types on plant water use efficiency and their mechanisms, a randomized block experiment was designed in the agro-pastoral ecotone of northern China during 2020–2021. The differences in dry matter accumulation, evapotranspiration, soil physical and chemical properties, soil water storage and water use efficiency and their relationships among cropland, natural grassland and artificial grassland were studied. The results show that: In 2020, the dry matter accumulation and water use efficiency of cropland were significantly higher than those of artificial and natural grassland. In 2021, dry matter accumulation and water use efficiency of artificial grassland increased significantly from 364.79 g·m^−2^ and 24.92 kg·ha^−1^·mm^−1^ to 1037.14 g·m^−2^ and 50.82 kg·ha^−1^·mm^−1^, respectively, which were significantly higher than cropland and natural grassland. The evapotranspiration of three land use types showed an increasing trend in two years. The main reason affecting the difference of water use efficiency was that land use type affected soil moisture and soil nutrients, and then changed the dry matter accumulation and evapotranspiration of plants. During the study period, the water use efficiency of artificial grassland was higher in years with less precipitation. Therefore, expanding the planted area of artificial grassland may be one of the effective ways to promote the full utilization of regional water resources.

## 1. Introduction

Water use efficiency is an index measuring the water absorption efficiency of plants, which is generally defined as the dry matter mass produced by water consumption per unit mass of plants [1]. As a broad concept, water use efficiency can be expressed in various ways in different research scales [2]. The main research scales include leaf level, population level, yield level and cell biology level. First, there are differences in water use efficiency between plants of different species [3,4]. In terms of crops, Wang et al. [5] analyzed four typical croplands in North America, Europe and Asia using flux data from 33 sites, and found that *Zea mays* had the strongest water use efficiency (2.48 g C·kg^−1^ H_2_O), followed by *Triticum aestivum* (2.00 g C·kg^−1^ H_2_O), *Glycine max* (1.92 g C·kg^−1^ H_2_O), and *Oryza sativa* (1.88 g C·kg^−1^ H_2_O). Similarly, there are differences in water use efficiency among different varieties of herbage, Xiong et al. [6] believed that due to significant differences in the net photosynthetic rate and transpiration rate of herbage as well as photosynthetic physiological changes in different seasons, the water use efficiency of *Trifolium pratense*, *Trifolium repens* and *Medicago sativa* was different, among which *Medicago sativa* had the highest water use efficiency, followed by *Trifolium repens*. *Trifolium pratense* has the lowest water use efficiency. Under the same grassland use intensity, the water use efficiency of different varieties of herbage was also different [7]. There are many reasons for the difference of water use efficiency among plants, including water use strategies of plants themselves [8] and the influence of external environment, such as soil water and soil nutrients [9,10]. Water is one of the main factors determining plant growth and development. Soil water deficit will lead to the reduction of stomatal conductance of plants, which will affect plant photosynthesis and transpiration. However, compared with transpiration, plant photosynthesis is less dependent on stomatal conductance, so moderate water deficit will promote the water use efficiency of plants [11]. The relationship between soil moisture and water use efficiency is also affected by climatic factors. Research has shown that with increasing saturated water vapor pressure difference, the sensitivity of water use efficiency to soil water content increased significantly. At low saturated water vapor pressure difference, water use efficiency is largely independent of soil water content [12]. Soil nutrients also play an important role in improving the water use efficiency of plants. Reasonable levels of nitrogen, phosphorus and potassium nutrients can improve the water relationship of plants to some extent, while at the same time improving the net photosynthetic rate of plants and the water use efficiency of individual leaves and population [13].

Land use type will affect soil moisture and soil nutrients, resulting in different water use efficiency. Huang et al. [14] showed that the continuous production of artificial grassland, such as *Medicago sativa*, will lead to serious soil water deficit and soil drying in arid areas as time goes by. Cropland has better water storage capacity [15], and may result in greater plant water use efficiency. However, the water use efficiency of cropland is greatly affected by soil water; under the condition of adequate soil water, the water use efficiency of cropland is at a high level, similar to that of forest land [16,17], while under the condition of insufficient soil water, the water use efficiency of cropland will be lower than that of artificial or natural grassland [18]. In addition to soil moisture, there are further differences in soil nutrients among different land use types [19]. Compared with grassland, cropland under artificial control has enough nutrients and water to ensure crop growth, so the water use efficiency of farmland may be higher than that of grassland [20]. Differences in land use type can indirectly affect the water use efficiency of plants, but there are many influencing factors, and the interaction among influencing factors cannot be ignored. Therefore, multiple factors should be comprehensively considered when analyzing the influencing factors of water use efficiency of different land use types.

The agro-pastoral ecotone refers to the semi agricultural and semi pastoral area in the transitional region between the semi humid agricultural region and the semi-arid grassland region in northern China [21], which has the characteristics of dramatic changes in land use. Liu et al. [22] showed that grassland, cropland, and forest land were the main types of land use in the northern agro-pastoral ecotone, and the total area was more than 4.30 × 10^5^ km^2^, accounting for more than 91.83% and the transfer area between grassland and cultivated land was as much as 1.67 × 10^4^ km^2^. In addition, as a sensitive zone of global climate change, the agro-pastoral ecotone is the most concentrated and obvious area of environmental problems [23]. A more prominent one is the contradiction of water use. Water shortages lead to a decline in regional ecosystem productivity, which becomes a key factor restricting regional agriculture and animal husbandry development [24]. Changes in land use type are one of the main forms by which human activities exert an influence on the natural environment. Such changes modify the structure, process and function of the ecosystem, and directly affect the hydrothermal balance of the ecosystem [25,26]. At present, most studies on water use efficiency focus on changing soil water and fertilizer conditions through field management measures to improve water use efficiency, yield, and economic benefits [27,28]. The above factors have been comprehensively considered, yet the research on the relationship between soil physical and chemical properties, water balance, and plant water use efficiency under different land use types is still weak. Therefore, it is of profound significance to explore the differences in water use efficiency of different land use types and their influencing factors in the agro-pastoral ecotone of northern China for the full utilization of water resources and the rational distribution of land in this area.

## 2. Results

### 2.1. Effects of Land Use Type on Soil Physical and Chemical Properties and Dry Matter Accumulation

**Figure 1:**
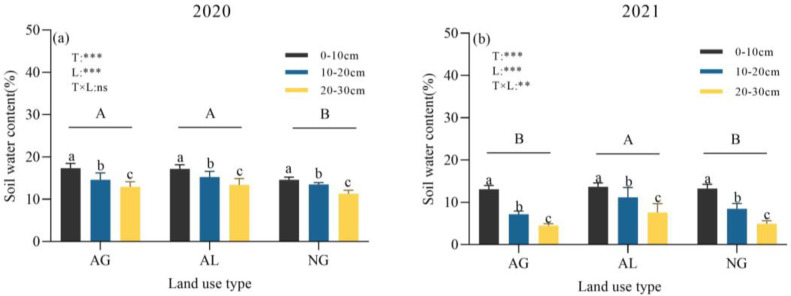
Soil volumetric water content at 0–10 cm, 10–20 cm, and 20–30 cm soil layers of different land use types in 2020 (**a**) and 2021 (**b**). AG: artificial grassland, AL: cropland, NG: natural grassland, T: land use type, L: soil layer, T × L: interaction of land use type and soil layer. Lowercase letters indicate significant differences between soil depths in the same treatment, and uppercase letters indicate significant differences between land use types (*p* < 0.05). ns, **, *** represent *p* > 0.05, *p* < 0.01, *p* < 0.001, respectively. Data are shown as mean ± s.e.m.

Land use type and soil layer in 2020 and 2021 had a significant influence on soil water content (*p* < 0.001), and the interaction between land use type and soil layer in 2021 had a significant influence on soil water content (*p* < 0.01, Figure 1a,b). In 2020, the soil moisture contents of the 0–10 cm, 10–20 cm and 20–30 cm layers in natural grassland were 14.60%, 13.53% and 11.31%, respectively, which are significantly lower than those of artificial grassland and cropland (Figure 1a). The soil water contents of 0–10 cm, 10–20 cm and 20–30 cm in the growing season of cropland in 2021 were 13.69%, 11.19% and 7.62%, respectively, which is significantly higher than those of artificial grassland and natural grassland (Figure 1b). With the increase in soil depth, soil water content decreased.

Soil organic carbon content was significantly affected by land use type, soil layer and year, and significantly affected by the interaction between land use type and soil layer, land use type and year, and soil layer and year (*p* < 0.05, Table S1) In 2020 and 2021, the contents of organic carbon and available phosphorus in 0–10 cm, 10–20 cm, and 20–30 cm soil tended to decrease with the increase in soil depth (Table 1). In 2020, soil organic carbon contents in the 0–10 cm, 10–20 cm and 20–30 cm soil layers of natural grassland were 12.34 g·kg^−1^, 11.06 g·kg^−1^ and 9.99 g·kg^−1^, respectively. This was significantly lower than those of artificial grassland and cropland in the same soil layer. In 2021, the soil organic carbon contents of natural grassland in the 0–10 cm, 10–20 cm and 20–30 cm soil layers were 14.62 g·kg^−1^, 11.59 g·kg^−1^ and 11.41 g·kg^−1^, respectively. This was also significantly lower than in cropland and artificial grassland. Soil available P content was significantly affected by land use type, soil layer and year (*p* < 0.05, Table S1). There were significant differences in 0–10 cm, 10–20 cm and 20–30 cm soil available phosphorus content among artificial grassland, cropland, and natural grassland in 2020 and 2021 (*p* < 0.05, Table 1). In 2020, the soil available phosphorus contents in the 0–10 cm, 10–20 cm and 20–30 cm soil layers of artificial grassland were 13.59 mg·kg^−1^, 9.89 mg·kg^−1^ and 8.31 mg·kg^−1^, respectively, which is significantly higher than those in cropland and natural grassland (*p* < 0.05, Table 1). In 2021, artificial grassland was also significantly higher than cropland and natural grassland (*p* < 0.05, Table 1).

The interaction of soil layer, year, land use type and soil layer significantly affected soil total P content (*p* < 0.05, Table S1). There was no significant difference in total phosphorus content in artificial grassland, cropland, and natural grassland in 2020 and 2021 (*p* > 0.05, Table 1). Soil total carbon content was significantly affected by land use type and year (*p* < 0.05, Table S1). In 2021, soil total carbon contents in the 0–10 cm and 20–30 cm soil layers of natural grassland were 20.23 g·kg^−1^ and 21.09 g·kg^−1^, respectively, which is significantly higher than in artificial grassland and cropland (*p* < 0.05, Table 1). Land use type, soil layer and year, as well as the interaction between land use type and soil layer, land use type and year had significant effects on soil total nitrogen (*p* < 0.05, Table S1). Soil total nitrogen contents in the 10–20 cm and 20–30 cm soil layers of natural grassland in 2020 were 1.17 g·kg^−1^ and 1.08 g·kg^−1^, respectively, which is significantly lower than those of artificial grassland and cropland (*p* < 0.05, Table 1). The difference in total nitrogen content among land use types in 2021 was as in 2020.

Soil NH_4_^+^-N, NO_3_^−^-N and available nitrogen contents were significantly affected by land use type and year, and soil nitrate nitrogen and available nitrogen contents were significantly affected by the interaction between land use type and year (*p* < 0.05, Table S1). There was no significant difference in soil NH_4_^+^-N content among artificial grassland, cropland, and natural grassland in 2020, and soil NH_4_^+^-N content of natural grassland in 2021 was 0.03 μg·cm^−2^·d^−1^, which was significantly lower than that of cropland. In 2020, the NO_3_^−^-N content of natural grassland was 0.68 μg·cm^−2^·d^−1^, which was significantly lower than that of artificial grassland and cropland. In 2021, soil NO_3_^−^-N content of natural grassland was 0.64 μg·cm^−2^·d^−1^, which was significantly lower than that of artificial grassland and cropland, and soil NO_3_^−^-N content of artificial grassland was 1.53 μg·cm^−2^·d^−1^, which was significantly lower than that of cropland. In terms of soil available nitrogen, the soil available nitrogen contents of cropland and artificial grassland in 2020 were 4.28 μg·cm^−2^·d^−1^ and 4.29 μg·cm^−2^·d^−1^, respectively, which is significantly higher than that of natural grassland. The soil available nitrogen content of cropland in 2021 was 2.73 μg·cm^−2^·d^−1^, which is significantly higher than those of artificial grassland and cropland (*p* < 0.05, Table 1).

**Figure 2:**
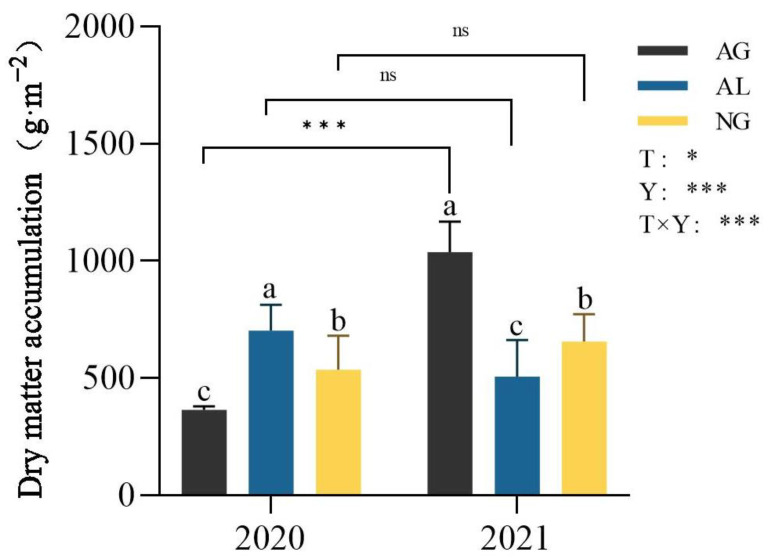
Dry matter accumulation between land use types in 2020 and 2021. AG: artificial grassland; AL: cropland; NG: natural grassland; T: land use type; Y: year; T × Y: interaction of land use type and year. Lowercase letters indicate the significant difference of dry matter accumulation among different land use types in the same year (*p* < 0.05). The asterisk indicates a significant difference between two years for the same land use type. *, ***, ns represent *p* < 0.05, *p* < 0.001, *p* > 0.05, respectively. Data are shown as mean ± s.e.m.

Dry matter accumulation was significantly affected by land use type, year, and their interaction (*p* < 0.05, Figure 2). In 2020, the dry matter accumulation of cropland was significantly higher than that of natural grassland and artificial grassland, and the dry matter accumulation of artificial grassland was 364.79 g·m^−2^, which was the lowest among the three land use types (*p* < 0.05, Figure 2). In 2021, dry matter accumulation of artificial grassland and natural grassland showed an increasing trend. Compared with 2020, the dry matter accumulation of artificial grassland and natural grassland in 2021 increased by 184.89% and 22.14% to 1037.14 g·cm^−2^ and 656.44 g·cm^−2^, respectively, while the dry matter accumulation of cropland decreased by 38.90% to 505.22 g·cm^−2^. Dry matter accumulation in artificial grassland was significantly higher than that in cropland and natural grassland (*p* < 0.05, Figure 2).

### 2.2. Water Storage, Evapotranspiration, and Soil Water Balance

Evapotranspiration, initial soil water storage, final soil water storage and soil water storage deficit degree were significantly affected by year and the interaction between year and land use type, soil water balance was significantly influenced by the interaction between year and land use type. (*p* < 0.05, Table S2). In 2020, compared with the initial soil water storage, the final soil water storage in artificial grassland, cropland, and natural grassland after the end of the growing season decreased by 11.8 mm, 1.39 mm, and 3.07 mm, respectively (Table 2). The final soil water storage capacity of cropland was 40.65 mm, significantly higher than that of artificial grassland and natural grassland (*p* < 0.05, Table 2). In 2021, the initial soil water storage capacity of 0–30 cm in natural grassland was 38.68 mm, which was significantly higher than those of artificial grassland and cropland (*p* < 0.05, Table 2). After the end of the growing season, the final soil water storage capacity of artificial grassland, cropland and natural grassland decreased by 3.55 mm, 5.70 mm (Table 2). There was no significant difference in evapotranspiration among land use types in 2020 (*p* > 0.05, Table 2). Evapotranspiration of natural grassland in 2021 was significantly higher than that of artificial grassland and cropland (*p* < 0.05, Table 2). Compared with 2020, evapotranspiration of artificial grassland, cropland and natural grassland showed an increasing trend in 2021 (Table 2). Soil water storage deficit degree of artificial grassland in 2020 was 65.65%, significantly higher than that of cropland (*p* < 0.05) and there was no significant difference in soil water storage deficit degree of artificial grassland, cropland, and natural grassland in 2021 (*p* > 0.05, Table 2). However, compared with 2020, soil water storage deficit of artificial grassland, cropland and natural grassland increased by 4.65%, 23.49% and 9.24%, respectively. Soil water balance of natural grassland in 2021 was significantly higher than those of artificial grassland and cropland (*p* < 0.05, Table 2).

### 2.3. Water Use Efficiency and Its Influencing Factors

**Figure 3:**
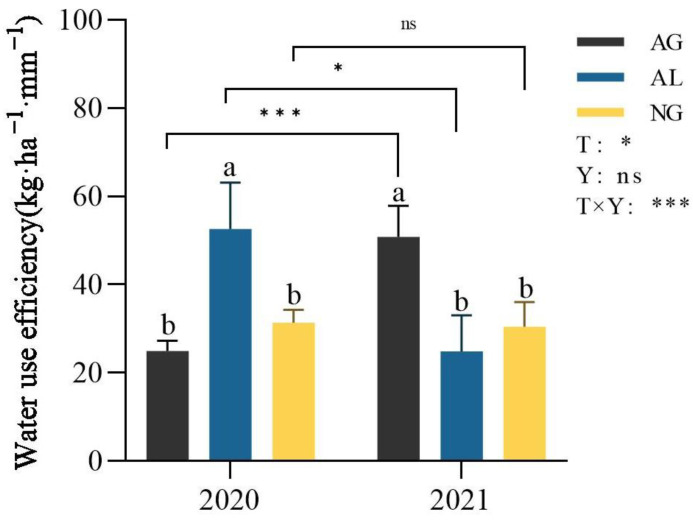
Water use efficiency of different land use types in 2020 and 2021. AG: artificial grassland; AL: cropland; NG: natural grassland; T: land use type; Y: year; T × Y: interaction of land use type and year. Lowercase letters indicate the significant difference between different land use type in the same year (*p* < 0.05). The asterisk indicates a significant difference between two years for the same land use type. *, ***, ns represent *p* < 0.05, *p* < 0.001, *p* > 0.05, respectively. Data are shown as mean ± s.e.m.

In 2020, the water use efficiency of cropland was 52.61 kg·ha^−1^·mm^−1^, which was significantly higher than those of artificial grassland and natural grassland (*p* < 0.05, Figure 3). In 2021, the water use efficiencies of cropland and natural grassland were reduced by 111.29% and 2.92%, respectively, while that of artificial grassland increased by 103.93% (Figure 3). The water use efficiency of artificial grassland was 50.82 kg·ha^−1^·mm^−1^·, which is significantly higher than those of cropland and natural grassland (*p* < 0.05, Figure 3). For the four plants planted in cropland and artificial grassland, the dry matter accumulation of AG1 and AG2 in 2020 was significantly lower than that of AL1 and AL2 (*p* < 0.01, Figure S1a), there was no significant difference in evapotranspiration among the four plants(*p* > 0.05, Figure S1b), and the water use efficiency of two plants in artificial grassland was significantly lower than that of two plants in cropland (*p* < 0.05, Figure S1c). In 2021, the dry matter accumulation of AG1 and AG2 was significantly higher than that of AL1 and AL2 (*p* < 0.01, Figure S1a), the change trend of water use efficiency and dry matter accumulation was consistent, and the water use efficiency of two plants in artificial grassland was significantly higher than that of two plants in cropland (*p* < 0.01, Figure S1c).

Comprehensive analysis of the relationship between soil physical and chemical properties, dry matter accumulation, evapotranspiration and water use efficiency at the growing season scale showed that, in 2020, soil water balance and nitrate nitrogen content had the greatest effect on the water use efficiency of artificial grassland (R^2^ = 0.994), initial soil water storage and dry matter accumulation had the greatest effect on the water use efficiency of cropland (R^2^ = 0.936), and soil total carbon content had the greatest effect on the water use efficiency of natural grassland (R^2^ = 0.994). In 2021, dry matter accumulation had the largest explanatory effect on WUE of natural grassland (R^2^ = 0.892), and dry matter accumulation and water balance had the largest explanatory effect on WUE of cropland and natural grassland (R^2^ = 0.999, R^2^ = 0.999). Combined 2020 and 2021, among the three land use types, dry matter accumulation and evapotranspiration constituted the largest explanations for water use efficiency of artificial grassland (R^2^ = 0.988), while evapotranspiration, dry matter accumulation and final soil water storage contributed the largest explanations for water use efficiency of cropland (R^2^ = 0.990). Dry matter accumulation, total nitrogen content and soil water balance had the highest explanatory degree for water use efficiency of natural grassland (R^2^ = 0.768, Table 3).

**Figure 4:**
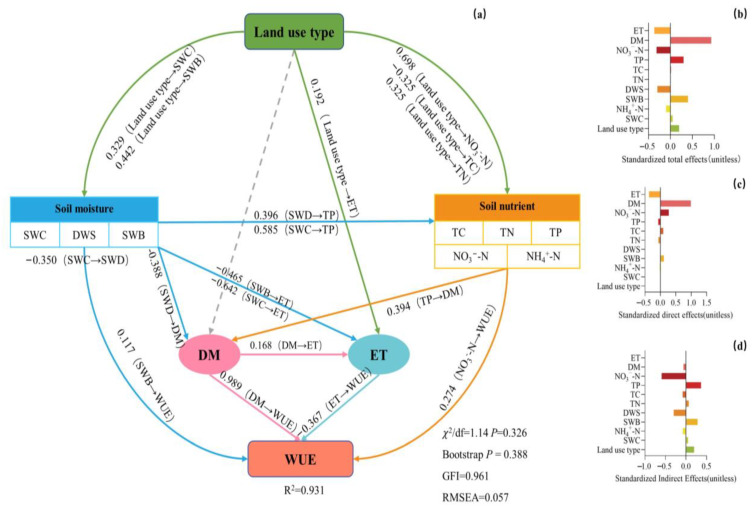
Structural Equation Model (SEM) describing the effect of land use types and environmental factors on water use efficiency. (**a**) Path analysis of influencing factors of water use efficiency, (**b**) standardized total effects of factors affecting water use efficiency. (**c**) Standardized direct effects of factors affecting water use efficiency. (**d**) Standardized indirect effects of factors affecting water use efficiency. Numbers adjacent to arrows are indicative of the effect size (*p* < 0.05) of the relationship. Grey dotted line indicates no significant relationship. R^2^ denotes the proportion of variance explained. Soil nutrients and soil moisture respectively contain several independent variables in the model, to simplify the graph, we group them in the same box in the model. TP: soil total phosphorus content; TC: soil total carbon content; TN: soil total nitrogen content; NH_4_^+^-N: soil ammonium nitrogen content; NO_3_^−^-N: soil nitrate nitrogen content; SWC: soil moisture content; DWS: soil water storage deficit degree; SWB: soil water balance; ET: evapotranspiration; DM: dry matter accumulation; WUE: water use efficiency. There was a non-significant deviation of the data from the model (χ2/df = 1.14; *p* = 0.326; Bootstrap P = 0.388; GIF = 0.961; RMSEA = 0.057).

Furthermore, the structural equation model was used to analyze the direct and indirect relationships between soil moisture, nutrients and water use efficiency in cropland and grassland in 2020 and 2021 growing seasons. Land use type directly affected evapotranspiration (direct effect 0.192) or soil water content (direct effect: 0.329), water balance (direct effect: 0.442), soil total carbon (direct effect: −0.325), soil total nitrogen (direct effect: 0.325) and NO_3_^−^-N content (direct effect: 0.698) indirectly affected dry matter accumulation and evapotranspiration, and thus affected plant water use efficiency. Soil water storage deficit degree could negatively regulate dry matter accumulation (direct effect: −0.388), soil total phosphorus content could positively regulate dry matter accumulation (direct effect: 0.394), soil water content and soil water balance could negatively regulate evapotranspiration (direct effect: −0.464 and −0.642). Dry matter accumulation can positively regulate evapotranspiration (direct effect: 0.168). Dry matter accumulation, soil water balance and soil NO_3_^−^-N content could directly positively regulate water use efficiency (direct effects were 0.989, 0.117 and 0.274), while evapotranspiration could directly negatively regulate water use efficiency (direct effects: −0.367, Figure 4a). In general, land use types affect plant dry matter accumulation and evapotranspiration directly or indirectly by affecting soil water content, water balance and soil nutrients, and ultimately affect plant water use efficiency (total effect is 0.197, indirect effect is 0.197, Figure 4b–d).

## 3. Discussion

### 3.1. Differences in Dry Matter Accumulation of Plants under Different Land Use Types

Dry matter accumulation of plants varies greatly under different land use types, which will affect the water use efficiency of plants to a certain extent. The results of this study showed that, in 2020, dry matter accumulation of cropland plants was higher than that of natural grassland and artificial grassland, and the artificial grassland was the lowest, only 364.79 g·m^−2^ (Figure 2). However, in 2021, dry matter accumulation of artificial grassland plants began to increase, even higher than that of cropland and natural grassland, which increased by 184.89% compared with the previous year (Figure 2). The reason for this may be that 2020 was the first year in which artificial grassland was planted, and only one harvest treatment was performed, resulting in low accumulation of plant dry matter. With the increase in the years of establishment of artificial grassland, the plant root system will gradually become stronger, improving its ability to extract more water from the soil [14,29]. Meanwhile, it also improves its ability to resist drought and other natural disasters, which enhances the vitality and adaptability of plants, and thus increases the accumulation of dry matter above ground and underground.

### 3.2. Differences of Soil Water Storage and Evapotranspiration under Different Land Use Types

Due to the characteristics of low precipitation, high evaporation intensity, and high groundwater depth, the agro-pastoral ecotone of north China is often in a state of soil water storage deficit, and there are certain differences in soil water storage and its deficit degree under different land use types [30,31]. The results of this study show that, compared with natural grassland and artificial grassland, cropland will have higher soil water storage capacity and correspondingly lower soil water storage deficit in 2020 (Table 2), which is consistent with the results of Shen and DuPont et al. [15,32]. However, there was no significant difference in evapotranspiration among land use types in 2020. In 2021, soil water storage deficit and evapotranspiration of grassland and cropland will both increase, which is because the precipitation in 2021 will decrease by 95 mm compared with that in 2020, and the initial soil water storage, final soil water storage and soil water storage deficit of cropland will increase significantly in 2021 (Table 2). The reason for this may be that the soil storage capacity of cropland is more susceptible to the influence of precipitation [33]. However, it is worth noting that the change in soil water storage deficit degree of artificial grassland was the least during 2020–2021 (Table 2), which may be because the successful construction of artificial grassland improves its ability to utilize deep soil water [32], indicating that artificial grassland has greater potential in soil water storage capacity.

### 3.3. Analysis of Differences and Influencing Factors of Plant Water Use Efficiency under Different Land Use Types

**Figure 5:**
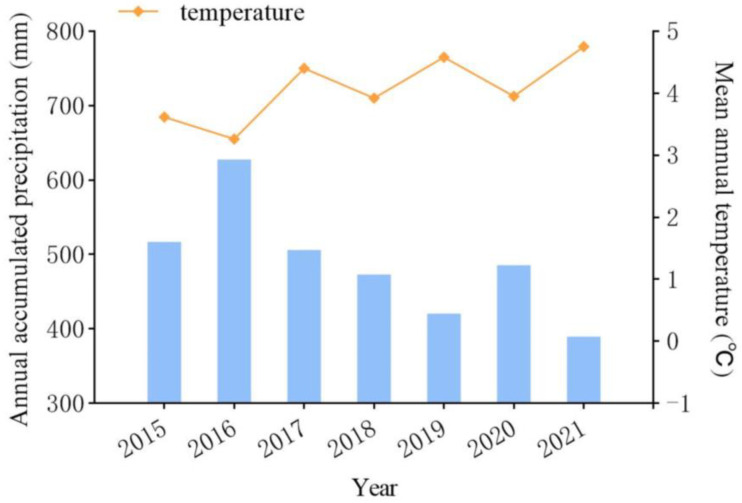
Annual precipitation and average annual temperature from 2015 to 2021.

In this study, plant water use efficiency was calculated by plant dry matter accumulation and evapotranspiration. The results showed that in 2020, plant water use efficiency of cropland was 52.61 kg·ha^−1^·mm^−1^, significantly higher than that of natural grassland and artificial grassland (Figure 3), which was consistent with the conclusion of Lei et al. [34]. However, compared with 2020, the water use efficiency of cropland and natural grassland in 2021 decreased by 111.29% and 2.92%, respectively, but the water use efficiency of artificial grassland increased by 103.93%, and the water use efficiency of artificial grassland was higher than that of cropland and natural grassland (Figure 3). The possible reasons include two aspects. First, the precipitation in 2020 was more sufficient than that in 2021, and the water storage capacity of cropland is already better than that of artificial grassland and natural grassland. Therefore, the water deficit degree of cropland was significantly lower than that of artificial grassland and natural grassland (Figure 5, Table 2). When the water is relatively sufficient, the accumulation of dry matter of cropland will be improved [35]. This may lead to increased water use efficiency in the plant population. Secondly, in order to ensure consistency with the tillage measures of residents in the study area and make the study more in line with the actual situation of the study area, seed fertilizer was applied in the field seeding, thus increasing the soil nutrient content, which would also lead to the increase in dry matter accumulation of plants [36,37]. Therefore, under the combined action of water and nutrients [38], the water use efficiency of cropland in 2020 was significantly higher than that of artificial grassland and natural grassland, which is the same as the results of Li and Kebebew et al. [39,40]. This is also consistent with our research results on the influence path of land use types on plant water use efficiency. Different land use types affect dry matter accumulation and evapotranspiration mainly through differences in soil nutrients and water, and thus affect water use efficiency (Figure 4). In 2021, the water use efficiency of artificial grassland was significantly higher than that of cropland and natural grassland (Figure 3). The reason for this may be that the number of mowing times of artificial grassland in 2021 increased from one to two times compared with that in 2020. Studies have shown that increasing the number of mowing times within a reasonable range will promote the increase in above-ground dry matter accumulation [41], indirectly leading to the improvement of water use efficiency, and at the same time, the decrease in farmland dry matter accumulation due to the decrease in precipitation will eventually lead to the decrease in water use efficiency. This is different from the results of Hou et al. [8], which may be due to the differences in climate conditions and soil nutrients and soil moisture among land use types in the study area, which will affect the dry matter accumulation and water consumption of plants [42,43], and ultimately lead to differences in water use efficiency among land use types. At the same time, due to the different measurement and calculation methods of water use efficiency, water use efficiency would be different, which would affect the evaluation of water use efficiency difference under different land use types. In the follow-up study, a variety of measurement methods for plant water use efficiency can be added to compare the effects of land use types on plant water use efficiency among different methods.

## 4. Materials and Methods

### 4.1. Site Description

**Figure 6:**
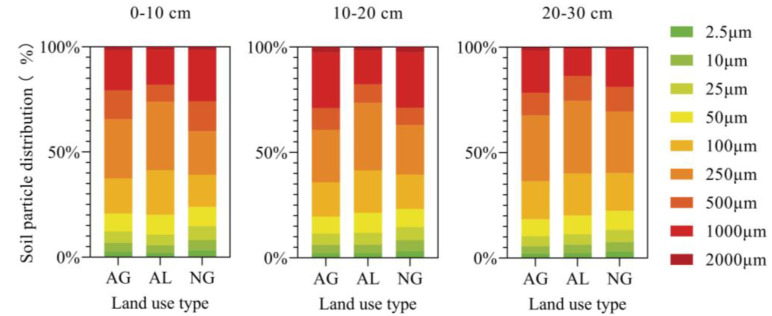
Soil particle distribution. AG: artificial grassland; AL: cropland; NG: natural grassland.

The experimental site is in Zhangjiakou Comprehensive Experimental Station of China Forage and Grass Research System in Zhangbei County, Hebei Province, China (41°28.7298′ N, 115°0.4562′ E), with an altitude of 1394 m. This region possesses a continental monsoon climate, cold, dry, windy, short frost-free period, sufficient light, day and night temperature difference. Precipitation is low and unevenly distributed, with the average annual precipitation ranging from 300 to 450 mm. During the study period, the annual precipitations in 2020 and 2021 were 485.70 mm and 389.80 mm, respectively, and the average annual temperatures were 3.95 ℃ and 4.75 ℃, respectively (Figure 5). The main type of soil is sandy chestnut soil, with shallow soil layer and poor soil (Figure 6). Grassland is typical steppe, with common plants including *Leymus chinensis*, *Stipa capillata*, *Agropyron cristatum*, *Artemisia argyi*, *Artemisia frigida*, etc., cropland planted plants include *Triticum aestivum*, *Avena chinensis*, *Avena sativa*, *Solanum tuberosum*, and *Sesamum indicum*, etc. Common artificial grassland includes *Medicago sativa* and *Bromus inermis*, etc.

### 4.2. Experimental Design and Field Management

**Figure 7:**
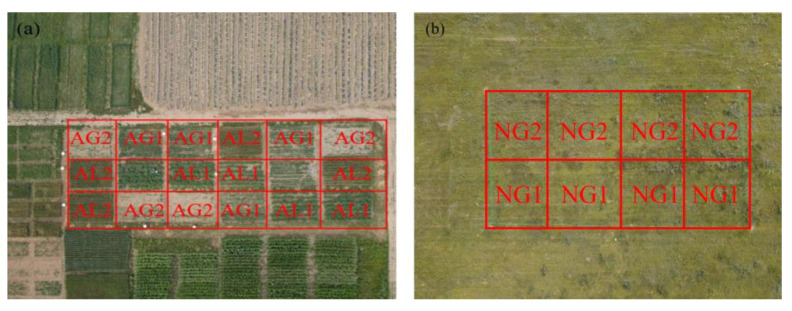
Map of the experimental site. (**a**) Artificial grassland and cropland. (**b**) Natural grassland AG1: *Medicago sativa*, AG2: *Bromus inermis*, AL1: *Solanum tuberosum*, AL2: *Avena sativa*, NG1: Enclosure, NG2: Cutting. The blank area in (**a**) is the installation and storage area of experimental equipment.

The field experiment started in May 2020, and three types of land use were selected as the research objects, including artificial grassland (AG), cropland (AL) and natural grassland (NG). The experimental designs of artificial grassland and cropland were randomized block design. Each treatment was configured with 4 replicates, and there were 16 plots in total. The plot area was 6 m × 8 m = 48 m^2^, and the total area was 768 m^2^ (Figure 7a). On 12–14 May, 2020, artificial grassland and cropland were planted. *Medicago sativa* (AG1) and *Bromus inermis* (AG2) were planted on artificial grassland, and *Solanum tuberosum* (AL1) and *Avena sativa* (AL2) were planted on cropland. The AL1 was hole seeded, and the other three plants were drill seeded. The seeding rate of AG1, AG2, AL1 and AL2 was 22.5 kg·ha^−1^, 30 kg·ha^−1^, 5 × 10^4^ stock·ha^−1^ and 225 kg·ha^−1^, respectively. No irrigation was applied to any of the land use types, and fertilizers were applied to cropland and artificial grassland at a rate of 750 kg·ha^−1^ compound fertilizer (N: P: K = 1:1:1) at sowing. The 2020 harvest time for cropland and artificial grassland was 30–31 Aug. In 2021, the sowing time of AL1 and AL2 was 10th May, the sowing method and fertilization method were the same as in 2020. After artificial grassland was successfully constructed, fertilization would not be applied. The harvest time of AL1 and AL2 in 2021 was 1st September and AG1 and AG2 were mowed twice, on 1st July and 1st August, to a stubble height of 7–8 cm. The total area of natural grassland test land was 30 m × 60 m = 1800 m^2^. It is divided into two treatments: enclosure (NG1) and cutting (NG2, Figure 7b). Cutting of NG2 was done in 1 September 2020 and 2021, to a stubble height of 7–8 cm.

### 4.3. Sampling and Measurements

In September 2020 and June to September 2021, one 1 m×1 m quadrat was randomly selected from each experimental plot to measure the aboveground dry matter mass of plants and calculate the accumulation of dry matter of plants in the plot. At the same time, 20 × 20 × 30 cm in situ soil was taken to obtain the mass of plant underground dry matter and used to calculate the accumulation of plant dry matter in the plot. At the end of plant sampling, the soil with a diameter of 5 cm was drilled to the depth of 0~10 cm, 10~20 cm and 20~30 cm. After drying and grinding, the soil samples were used to determine the soil nutrient content. The contents of total nitrogen and total carbon in soil were analyzed by an element analyzer (Vario Max CN; Elementar, Hanau, Germany) for determination. The contents of total phosphorus and available phosphorus in soil were determined by molybdenum-antimony colorimetry. Soil organic carbon content was determined by wet oxidation using K_2_Cr_2_O_7_ and H_2_SO_4_ [44]. Soil available nitrogen content was identified using in situ, nondestructive ion exchange membranes (Ionics, Ringwood, NJ, USA). In 2020 and 2021, portable soil moisture sensors (STVNS Hydra Probe soil moisture sensor, the accuracy is ±0.03%) were used to measure the soil moisture content (%) at 0–10 cm, 10–20 cm, and 20–30 cm soil layers in each plot once every 7 days from June to August.

### 4.4. Soil Moisture and Water Use Efficiency Index Calculation

The calculation formula of soil water storage (SWS) is as follows [45]:

(1) $$SWS=\left(SD\times R\times Wm\right)\times 10$$

where SWS, SD, R and Wm respectively represent soil water storage (mm), soil depth (cm), soil bulk density (g·cm^−3^) and soil mass water content (%) at a given depth.

Soil water storage deficit degree (DSW) was calculated according to the following equations [14]:

(2) Da=Fc−SWS

(3) $$DWS=\left(Da/Fc\right)\times 100$$

where Da is soil water storage deficit (mm) and Fc is field capacity (in this study, it was 84.04 mm for cropland and artificial grassland, and 77.88 mm for natural grassland).

The ET (mm) was determined for each year of the study according to the following equations [45]:

(4) ET=P+ΔS

where P is the effective precipitation of the growing season (≥5 mm) [46], ΔS is the change in soil water storage in the profile of research depth (mm).

Since the test was conducted under rain-nourished conditions, irrigation was not carried out on the plot and there was no surface runoff. Meanwhile, since the underground water depth was greater than 50 m, the capillary rise and drainage in the root zone and deep percolation were also considered to be negligible.

The water use efficiency (WUE, kg·ha^−1^·mm^−1^) was determined for each year of the study according to the following equations [47]:

(5) WUE=Y/ET

where Y is the accumulation of plant dry matter (g·m^−2^).

### 4.5. Statistical Analysis

Two-way and multi-factor ANOVA analysis and multiple comparison of all data were performed using SPSS (version 16.0, SPSS, Chicago, IL, USA). Duncan’s significant difference test was used to compare the mean values (*p* < 0.05) and conducted stepwise regression analysis on the relationship between water use efficiency and soil physicochemical properties, water content, dry matter accumulation and evapotranspiration. SPSS-Amos was used to analyze the influence paths of water resources use efficiency of different land use types.

## 5. Conclusions

The study on plant water use efficiency and its influencing factors of different land use types (cropland, natural grassland, artificial grassland) in the agro-pastoral ecotone in northern China showed that during the study period, the dry matter accumulation and soil water content of cropland plants were higher than that of natural grassland and artificial grassland in the year with more precipitation (2020). However, in the year with less precipitation (2021), the change in soil water storage of artificial grassland was low, and with the increase in planting years and mowing times, the accumulation of plant dry matter also increased. Therefore, the water use efficiency of artificial grassland was higher than that of cropland and natural grassland. The results of this study provide an important theoretical basis for the full utilization of soil and water resources in the northern farming-pastoral ecotone in years with different precipitation.

## Figures and Tables

**Table 1:** Soil nutrient index in the 0–10 cm, 10–20 cm and 20–30 cm soil layers for different land use types in 2020 and 2021.

		AG	AL	NG	AG	AL	NG	AG	AL	NG
		0~10 cm			10~20 cm			20~30 cm		
2020	TP (g·kg^−1^)	0.29 ± 0.03	0.27 ± 0.02 A	0.21 ± 0.03	0.23 ± 0.03	0.22 ± 0.02 AB *	0.20 ± 0.02	0.21 ± 0.03	0.18 ± 0.03 B	0.19 ± 0.02
	TC (g·kg^−1^)	15.67 ± 0.34	17.16 ± 0.88	17.66 ± 0.53	16.18 ± 0.41	16.77 ± 0.87	15.84 ± 0.44	15.71 ± 0.37	16.44 ± 1.17	17.91 ± 0.61
	TN (g·kg^−1^)	1.60 ± 0.08 *	1.72 ± 0.05	1.47 ± 0.09 A	1.81 ± 0.05 a *	1.65 ± 0.05 a	1.17 ± 0.06 bB	1.66 ± 0.09 a *	1.63 ± 0.08 a	1.08 ± 0.07 bB
	SOC (g·kg^−1^)	14.72 ± 0.32 a	13.90 ± 0.39 abA	12.34 ± 0.59 bA	14.38 ± 0.36 a **	14.38 ± 0.29 aA	11.06 ± 0.23 bAB	13.86 ± 0.28 a	12.84 ± 0.43 aB *	10.00 ± 0.33 bB
	Olsen-P (mg·kg^−1^)	13.59 ± 1.74 aA	10.44 ± 0.70 abA	8.45 ± 0.40 Ba *	9.90 ± 0.73 bA *	8.03 ± 0.76 abB **	6.85 ± 0.60 bAB *	8.31 ± 0.51 aB **	5.17 ± 0.50 bC	5.43 ± 1.06 bB *
	NH_4_^+^-N (μg·cm^−2^·d^−1^)	0.11 ± 0.03	0.07 ± 0.02	0.02 ± 0.01	-	-	-	-	-	-
	NO_3_^−^-N (μg·cm^−2^·d^−1^)	4.19 ± 0.17 a ***	4.21 ± 0.14 a **	0.68 ± 0.03 b	-	-	-	-	-	-
	Available N (μg·cm^−2^·d^−1^)	4.29 ± 0.17 a ***	4.28 ± 0.16 a **	0.70 ± 0.03 b	-	-	-	-	-	-
2021	TP (g·kg^−1^)	0.26 ± 0.02 A	0.21 ± 0.03	0.18 ± 0.03	0.19 ± 0.02 B	0.13 ± 0.02	0.20 ± 0.02	0.16 ± 0.02 bB	0.14 ± 0.02 b	0.22 ± 0.02 a
	TC (g·kg^−1^)	16.19 ± 0.93 b	17.26 ± 0.89 b	20.23 ± 0.50 a	16.67 ± 1.33	17.03 ± 0.90	18.82 ± 0.38	15.76 ± 1.11 b	17.35 ± 1.18 b	21.09 ± 0.88 a
	TN (g·kg^−1^)	1.49 ± 0.06	1.58 ± 0.06	1.51 ± 0.03 A	1.48 ± 0.07 a	1.53 ± 0.08 a	1.27 ± 0.03 bB	1.41 ± 0.07 a	1.51 ± 0.08 a	1.09 ± 0.03 bC
	SOC (g·kg^−1^)	15.05 ± 0.32 A	15.49 ± 0.35	14.62 ± 0.30 A	12.98 ± 0.18 bC	14.53 ± 0.60 a	11.60 ± 0.16 cB	13.12 ± 0.38 bB	14.92 ± 0.31 a	11.41 ± 0.78 cB
	Olsen-P (mg·kg^−1^)	12.90 ± 3.87 a	10.16 ± 2.20 abA	3.38 ± 0.21 b	7.49 ± 1.04 a	4.44 ± 0.31 bB	2.88 ± 0.21 b	5.93 ± 0.48 a	4.59 ± 1.07 abB	2.73 ± 0.26 b
	NH_4_^+^-N (μg·cm^−2^·d^−1^)	0.04 ± 0.01 ab	0.05 ± 0.01 a	0.03 ± 0.01 b	-	-	-	-	-	-
	NO_3_^−^-N (μg·cm^−2^·d^−1^)	1.53 ± 0.14 b	2.68 ± 0.31 a	0.64 ± 0.05 c	-	-	-	-	-	-
	Available N (μg·cm^−2^·d^−1^)	1.57 ± 0.14 b	2.73 ± 0.31 a	0.67 ± 0.05 c	-	-	-	-	-	-

TP: soil total phosphorus content; TC: soil total carbon content; TN: soil total nitrogen content; SOC: soil organic carbon content; Olsen-P: soil available phosphorus content; NH_4_^+^-N: soil ammonium nitrogen content; NO_3_^−^-N: soil nitrate nitrogen content; Available N: soil available nitrogen content; AG: artificial grassland; AL: cropland; NG: natural grassland. Lowercase letters indicate the significant difference between different land use type in the same year and soil depth, capital letters indicate significant differences between different soil layers under the same land use type and year (*p* < 0.05). The asterisk indicates a significant difference between two years for the same land use type and soil layer. *, **, *** represent *p* < 0.05, *p* < 0.01, *p* < 0.001, respectively. Data are shown as mean ± s.e.m.

**Table 2:** Soil water storage, soil water balance, storage deficit degree in the 0–30 cm soil depth and evapotranspiration at different land use type in 2020 and 2021.

Year	Land Use Type	Initial Soil Water Storage (mm)	Final Soil Water Storage (mm)	Evapotranspiration (mm)	Soil Water Storage Deficit Degree (%)	Soil Water Balance (mm)
2020	AG	40.75 ± 3.89	28.95 ± 3.29 b	146.10 ± 3.11 ***	65.56 ± 3.92 a	−11.80 ± 3.11
	AL	42.04 ± 4.46 *	40.65 ± 3.00 a**	135.70 ± 5.39 ***	51.64 ± 3.58 b**	−1.40 ± 5.39
	NG	31.53 ± 2.54 *	28.46 ± 3.02 b	137.38 ± 0.97 ***	63.46 ± 3.88 ab	−3.08 ± 0.97
2021	AG	28.59 ± 3.43 b	25.04 ± 3.00	201.95 ± 1.87 b	70.21 ± 3.56	−3.55 ± 1.87 a
	AL	26.60 ± 2.47 b	20.90 ± 2.09	204.10 ± 2.97 b	75.13 ± 2.49	−5.7 ± 2.97 a
	NG	38.68 ± 2.80 a	21.26 ± 1.82	215.81 ± 3.19 a	72.70 ± 2.34	−17.41 ± 3.19 b

AG: artificial grassland; AL: cropland; NG: natural grassland. Lowercase letters indicate the significant difference among different land use types in the same year (*p* < 0.05). The asterisk indicates a significant difference between two years for the same land use type. *, **, *** represent *p* < 0.05, *p* < 0.01, *p* < 0.001, respectively. Data are shown as mean ± s.e.m.

**Table 3:** Stepwise regression analysis of water use efficiency and soil physicochemical properties, dry matter accumulation and evapotranspiration.

Year	Land Use Type	Equation	*p*	R^2^
2020	AG	WUE = 22.38 + 0.38SWB + 1.68NO_3_^−^ − N	**	0.994
	AL	WUE = 38.35 − 0.46ISW + 0.05DM	**	0.936
	NG	WUE = 81.63 − 2.79TC	*	0.994
2021	AG	WUE = −3.00 + 0.05DM	***	0.892
	AL	WUE = 1.00 + 0.05DM + 0.12SWB	***	0.999
	NG	WUE = 2.49 + 0.05DM + 0.13SWB	***	0.999
2020–2021	AG	WUE = 28.68 + 0.05DM − 0.16ET	***	0.988
	AL	WUE = 108.31 − 0.46ET + 0.04DM − 0.46FSW	***	0.990
	NG	WUE = 54.94 + 0.03DM − 27.51TN + 0.23SWB	***	0.768

WUE: water use efficiency; DM: dry matter accumulation; ET: evapotranspiration; FSW: final soil water storage; ISW: initial soil water storage; TN: soil total nitrogen content; TC: soil total carbon content; SWB: soil water balance; NO_3_^−^-N: soil nitrate nitrogen content; AG: artificial grassland; AL: cropland; NG: natural grassland. *, **, *** represent *p* < 0.05, *p* < 0.01, *p* < 0.001, respectively.

## Data Availability

Not applicable.

## Supplementary Materials

The following supporting information can be downloaded at: https://www.mdpi.com/article/10.3390/plants12061239/s1, Table S1: Results of multi-factor ANOVA analysis showing the effects of land use type(T), soil layer(L) and year(Y) and their interactions on soil physical and chemical properties; Table S2: Results of two-way ANOVA analysis showing the effects of land use type(T) and year(Y) and their interaction on soil moisture conditions; Figure S1: Differences of dry matter accumulation(a), evapotranspiration(b), and water use efficiency(c) between different plants in cropland and artificial grassland in 2020–2021.
