# Supplementary material for: Artificial Grassland Had Higher Water Use Efficiency in Year with Less Precipitation in the Agro-Pastoral Ecotone
Source: Plants (Basel). 2023 Mar 9;12(6):1239. doi: 10.3390/plants12061239 (PMC10059974; doi:10.3390/plants12061239)
Supplement: Supplementary file 1 [file plants-12-01239-s001.zip › plants-2251615-supplementary.pdf]

Table S1. Results of multi-factor ANOVA analysis showing the effects of land use type(T), soil layer(L) and year(Y) and their interactions on soil physical and chemical properties

	T		L		Y		T×L		T×Y		L×Y		T×L×Y	
	F	*P*	F	*P*	F	*P*	F	*P*	F	*P*	F	*P*	F	*P*
SWC	27.06	***	197.86	***	511.65	***	2.40	0.05	13.27	***	21.4<br>3	***	2.17	0.08
Olsen-P	20.44	***	19.27	***	15.85	***	1.83	0.13	1.58	0.21	0.58	0.56	0.58	0.68
SOC	56.41	***	25.40	***	12.44	**	3.55	**	11.82	***	6.13	**	0.65	0.63
TC	9.41	***	0.48	0.62	7.01	**	1.09	0.36	2.83	0.06	0.04	0.96	0.08	0.99
TN	37.65	***	8.55	***	9.27	**	5.13	**	5.57	**	0.22	0.80	0.65	0.63
TP	2.46	0.09	7.38	**	9.82	**	3.01	*	2.96	0.06	0.47	0.63	0.47	0.76
NH_4^+-N	3.50	*	-	-	4.28	*	-	-	1.89	0.17	-	-	-	-
NO_3^--N	107.88	***	-	-	82.57	***	-	-	22.15	***	-	-	-	-
Available N	109.87	***	-	-	85.12	***	-	-	23.04	***	-	-	-	-

SWC: soil moisture content, Olsen-P: soil available phosphorus content, SOC: Soil organic carbon content, TC: soil total carbon content, TN: Soil total nitrogen content, TP: soil total phosphorus content, NH_4^+-N: soil ammonium nitrogen content, NO_3^--N: soil nitrate nitrogen content, Available N: soil available nitrogen content. The asterisk indicates a significant difference between two years for the same land use type and soil layer. *,**,*** represent $P < 0.05$, $P < 0.01$, $P < 0.001$,respectively.

Table S2. Results of two-way ANOVA analysis showing the effects of land use type(T) and year(Y) and their interaction on soil moisture conditions.

	T		Y		T×Y	
	F	*P*	F	*P*	F	*P*
Initial soil water storage	0.02	0.98	5.22	*	4.91	*
Final soil water storage	2.15	0.13	19.80	***	4.81	*
Evapotranspiration	1.70	0.20	518.35	***	4.68	*
Soil water storage deficit degree	1.28	0.29	19.47	***	4.61	*
Soil water balance	1.70	0.20	1.36	0.25	4.68	*

The asterisk indicates a significant difference between two years for the same land use type. *,*** represent $P < 0.05$, $P < 0.001$,respectively.

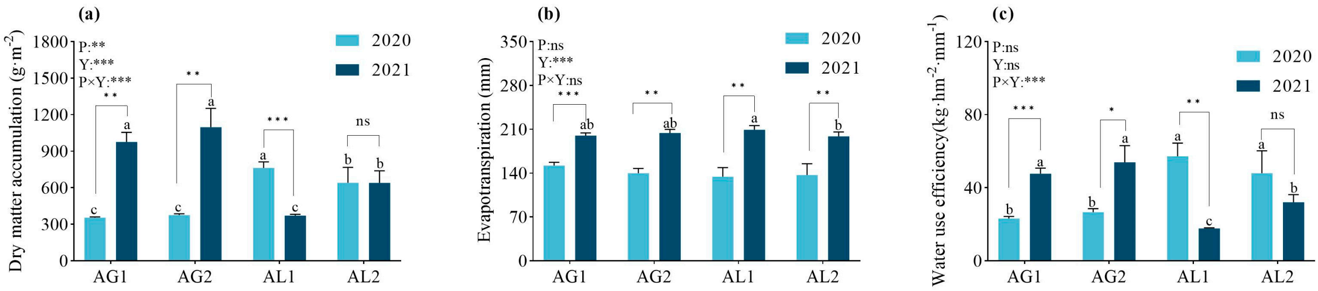

Figure S1. Differences of dry matter accumulation(a), evapotranspiration(b), and water use efficiency(c) between different plants in cropland and artificial grassland in 2020-2021. AG1: *Medicago sativa*, AG2: *Bromus inermis*, AL1: *Solanum tuberosum*, AL2: *Avena sativa*. P: plant, Y: year, P×Y: interaction of plant and year. Lowercase letters indicate the significant difference between different plant in the same year ($P < 0.05$). The asterisk indicates a significant difference between two years for the same plant. *, **, ***, ns represent $P < 0.05$, $P < 0.01$, $P < 0.001$, $P > 0.05$, respectively. Data are shown as mean ± s.e.m.
